# Intraoperative electromyographic monitoring to optimize safe lumbar pedicle screw placement – a retrospective analysis

**DOI:** 10.1186/s12891-017-1594-1

**Published:** 2017-05-30

**Authors:** Arun-Kumar Kaliya-Perumal, Jiun-Ran Charng, Chi-Chien Niu, Tsung-Ting Tsai, Po-Liang Lai, Lih-Huei Chen, Wen-Jer Chen

**Affiliations:** 1 Department of Orthopaedic Surgery, Spine Division, Bone and Joint Research Center, Chang Gung Memorial Hospital and Chang Gung University College of Medicine, No. 5, Fusing St., Gueishan, Taoyuan, 333 Taiwan; 2 Department of Orthopaedic Surgery, Melmaruvathur Adhiparasakthi Institute of Medical Sciences and Research, Melmaruvathur, Tamil Nadu India

**Keywords:** Electromyography, Neuromonitoring, Neurologic manifestations, Neurophysiology, Pedicle screws

## Abstract

**Background:**

The foremost concern of a surgeon during pedicle screw fixation is safety. Assistive modalities, especially intraoperative electromyographic monitoring (EMG) can function as an essential tool to recognize screw malposition that compromise neural integrity, so that the screws can be repositioned immediately rather than later. We intend to study the efficacy of intraoperative EMG monitoring to detect potential pedicle breach and evaluate whether reoperation rates were significantly reduced.

**Methods:**

Retrospectively, patients who underwent posterior stabilization with pedicle screws for various pathologies were analysed and those with screws among L1-S1 levels were shortlisted. They were divided into two groups. Group 1 included patients in whom trigger EMG (t-EMG) was used to confirm appropriate screw placement and Group 2 included those in whom it was not used. Responses to t-EMG and corresponding stimulation thresholds were recorded for Group 1 patients. The sensitivity and specificity of the test was calculated. Reoperation rates due to postoperative neurologic compromise caused by malpositioned screws were compared between both the groups.

**Results:**

A total of 518 patients had 3112 pedicle screws between L1-S1 levels. Among Group 1 [*n* = 296; Screws = 1856], 145 screws (7.8%) showed a positive response for t-EMG at stimulation thresholds ranging between 2.6 to 19.8 mA. The sensitivity and specificity of t-EMG to diagnose potential pedicle breach was found to be 93.33% and 92.88% respectively. Only one patient among Group 1 required reoperation. However, among Group 2 [*n* = 222; screws = 1256], six patients required reoperation. This indicated a significant decrease in the number of malpositioned screws that caused neurological compromise [*p* = 0.02], leading to subsequent decrease in reoperation rates [*p* = 0.04] among Group 1 patients.

**Conclusions:**

Trigger EMG is well efficient in detecting potential pedicle screw breaches that might endanger neural integrity. In combination with palpatory and radiographic assessment, it will certainly aid safe and secure pedicle screw placement. It can also efficiently reduce reoperation rates due to neurologic compromise provoked by a malpositioned screw.

## Background

Utmost care needs to be taken during pedicle screw placement to prevent potential damage to neural structures that closely approximate the bony pedicle. Pedicle screw technique has evolved over time and experience; however, “free-hand” technique is most widely practiced [1–5]. This technique involves visual identification of certain anatomical landmarks to find an appropriate entry point and then preparing the pedicle for screw placement [6, 7]. Whether the instruments used or the screw itself had breached the pedicle, is completely dependent on surgeon’s perception. Hence, considerable expertise is required to safely perform this technique.

Even though, intraoperative confirmation of secure screw placement can be obtained using fluoroscopic images, neural integrity or compromise needs to be checked. Moreover, completely depending on fluoroscopic images may not be appropriate, especially when proper anatomy cannot be visualized. Therefore, we believe that intraoperative electromyographic monitoring (EMG) can be an essential tool to recognize screw malposition that compromise neural integrity [8, 9]. Such detection gives a warning so that the screws can be repositioned immediately rather than later [10]. We intend to study the efficacy of intraoperative EMG monitoring to detect potential pedicle breach during free-hand lumbar pedicle screw placement and evaluate whether reoperation rates were significantly reduced.

## Methods

We retrospectively reviewed data of all patients who underwent posterior instrumentation with pedicle screw constructs for various pathologies at our institution over a period of time. Their preoperative x-rays and MRI were evaluated. The indications of surgery included:

- Degenerative spondylosis,
- Spondylolisthesis (≥ grade 1),
- Spondylodiscitis and
- Tumours.

Only those patients with pedicle screws in L1-S1 levels were shortlisted and a sample containing 518 patients was obtained. They were divided into two groups based on intraoperative EMG monitoring. EMG monitoring was utilized based on the availability of neuromonitoring system and the operating personnel. Those patients in whom intraoperative EMG was used were Group 1 (Experimental group) and the remaining patients were Group 2 (Control group).

We excluded:

- Those patients with dysmorphic pedicle features, such as in congenital scoliosis,
- Those in whom minimally invasive percutaneous screw insertion was performed and
- Those with pedicle screws in vertebrae other than L1-S1.

Surgeries were performed by five experienced orthopaedic surgeons and their procedure to apply pedicle screw was unanimous. Although, we do both open surgery with free hand technique and minimally invasive surgery (MIS) with percutaneous screw insertion under fluoroscopic guidance, we only included patients in whom open surgery with free hand technique was used to place the screws. All surgeons identified bony landmarks including pars, transverse process and facet joints before making the entry point. Partial infero-lateral corticotomy of the superior facet was performed in line with the mid-point of transverse process to identify the appropriate entry point. Following entry, the pedicle was probed and walls were felt using a pedicle sound to assess structural integrity. If satisfactory tactile perception was obtained, pedicle screws were placed. This technique of pedicle screw placement was customary during the study period.

Group 1 patients had recording electrodes placed in muscles of interest; hence, handling of nerve roots may produce spontaneous response in the corresponding muscle. However, this recording cannot be continuous due to the use of muscle relaxants for anaesthesia; therefore, trigger EMG (t-EMG) was used to identify nerve root compromise following screw placement. Neuromuscular blocking agents were not used for at least 30 min prior to testing t-EMG. Disposable pedicle screw stimulating probes (Cadwell Laboratories Inc., Kennewick, WA, USA) which had a fully long insulated shaft terminating in a three millimetre ball tip was used to pass an electrical stimulus of up to 20 mA to the screw. Electrophysiological monitoring was conducted using Cascade Elite Intra-Operative Neuro-Monitoring (IONM) system (Cadwell Laboratories Inc., Kennewick, WA, USA). A positive response below 20 mA was considered as pedicle breach and a negative response was considered as accurately positioned. A positive response alerts the surgeon to attempt direct visualization of the medial pedicle wall. If the screw had violated the medial cortex, they were either revised or removed based on surgeon’s decision. Screw placement was also checked intraoperatively using x-ray images after completing the construct.

Group 2 patients had pedicle screws applied with similar technique except for the use of EMG. Screw positioning was checked only by means of intraoperative x-ray image after completing the construct. Post-operative X-rays were taken for all patients among both the groups. Further evaluation by CT scanning was arranged if there was clinical evidence of neurological compromise. If malpositioning was determined by post-operative CT evidence, reoperation was performed to revise or remove the screw. Patient demographics, clinical presentation, indications for operation and operative variables were reviewed for each case. The efficacy of intraoperative EMG monitoring to identify screw breach and subsequent decrease in reoperation rates were evaluated.

Statistical analysis was performed using SPSS software v.18.0 for Windows (SPSS Inc., Chicago, IL, USA). Student’s t-test was used for continuous variables and Fisher’s exact test for categorical variables. A probability (*p*) value of <0.05 was considered statistically significant.

## Results

We shortlisted 518 patients [mean age ± standard deviation (SD) = 63.6 ± 11.5 years; 3112 pedicle screws] based on our inclusion criteria. Patients who received EMG monitoring throughout the procedure and t-EMG to determine screw placement were in Group 1 [*n* = 296; mean age = 64.2 ± 11.7; Screws = 1856]. The remaining patients who did not receive EMG monitoring were in Group 2 [*n* = 222; mean age = 62.9 ± 11.3; screws = 1256]. Demographic comparison between both groups was tabulated (Table 1). The groups were statistically similar in age [*p* = 0.17] and gender distribution [*p* = 1]; however, the overall number of screws and the number of screws per patient was more in Group 1.

**Table 1:** Demographic Data

Variables	Group 1	Group 2
No. of patients	*n* = 296 [Male = 106; Female = 190]	*n* = 222 [Male = 80; Female = 142]
Age	64.2 ± 11.7 years	62.9 ± 11.3 years
Total No. of pedicle screws	1856	1256
No. of screws per patient	6.3 ± 2	5.6 ± 2
No. of screws in L1	40	14
No. of screws in L2	162	64
No. of screws in L3	384	196
No. of screws in L4	542	396
No. of screws in L5	548	432
No. of screws in S1	180	154

Among 1856 screws in Group 1, 145 screws (7.8%) showed a positive response for t-EMG at stimulation thresholds ranging between 2.6 to 19.8 mA. Number of screws showing positive response at various threshold ranges was tabulated (Table 2). Even though 145 screws showed positive response, only 14 had actually breached the medial pedicle wall. This was determined based on intraoperative assessment by direct visualization of the medial cortex and X-ray images. Hence, there were 14 true positive (9.7%) and 131 false positive responses (90.3%). Various stimulation threshold ranges corresponding to true positive responses were tabulated (Table 3).

**Table 2:** Overall number of screws that recorded positive response at various stimulation threshold ranges

No. of screws showing positive response	Stimulation threshold
6	<5 mA
26	5–10 mA
45	10–15 mA
68	15–20 mA

**Table 3:** Number of screws that recorded positive response which were true positive

No. of patients showing positive response	Stimulation threshold
3	<5 mA
9	5–10 mA
2	10–15 mA
0	15–20 mA

The remaining 1711 screws (92.2%) showed no response to t-EMG. However, one patient among those who had negative response for t-EMG, who had received posterior instrumentation from L2-L5, presented with postoperative clinical evidence of neurologic compromise. Postoperative CT scanning showed that one of the L2 screws had more than 25% of its diameter residing outside the medial pedicle wall, which required a revision surgery. The corresponding t-EMG recording was considered as a false negative response. This observation made us to analyse the sensitivity and specificity of t-EMG to diagnose potential pedicle breach, and was found to be 93.33% and 92.88% respectively (Table 4).

**Table 4:** – Sensitivity and specificity analysis

t-EMG Monitoring	Breached Pedicle (*n*)	Intact Pedicle (*n*)	Total
Positive	True positive = 14	False positive = 131	145
Negative	False Negative = 1	True Negative = 1710	1711
Total	15	1841	1856
Sensitivity	93.33%		
Specificity	92.88%		

For those in Group 2 where EMG was not used, surgeon’s tactile perception and intraoperative X-ray images were the only modalities to assess screw placement. Even though satisfactory screw placement was achieved intraoperatively, six patients had postoperative clinical evidence of neurologic compromise and CT scanning was done. Each of these patients had one screw that was identified to have breached the medial pedicle wall that prompted revision surgery. Our overall results indicated a significant decrease in the number of malpositioned screws that caused neurological compromise [*p* = 0.02], leading to subsequent decrease in reoperation rates [*p* = 0.04] among Group 1 patients (Table 5).

**Table 5:** – Results

Variable	Group 1	Group 2	Statistical analysis
No. of malpositioned screws causing neurologic compromise	1	6	*p =* 0.02^*^
No. of patients who required reoperation	1	6	*p =* 0.04^*^

*probability value “*p*” less than 0.05 was considered statistically significant

## Discussion

Malpositioning of pedicle screws can lead to various complications (8). Screws breaching the pedicle pose a potential threat to adjacent neural structures and the most alarming consequence is neurological deficit. However, this can be avoided by meticulous technique. Recent advances in image guided navigation modalities that aid the surgeon to perform safe pedicle screw placements are gaining popularity [11, 12]. Yet, intraoperative neuromonitoring using continuous EMG and t-EMG are widely accepted as standard modalities [8, 13].

Continuous EMG monitoring acts as a real time monitor of spontaneous activity [14]. Any irritation to the nerve by stretch or compression causes trains of motor unit potential discharge in the corresponding muscle [14]. But the use of short acting neuromuscular blocking agents, interferes with this recording [15]. It is for this reason, neuromuscular blocking agents were not administered for at least 30 min prior to testing t-EMG. We stimulated the conductive pedicle screw head up to 20 mA and checked for response. A positive response at a relatively low threshold suggests definite malposition of the screw, violating the pedicle wall [10, 14, 16, 17].

Our analysis showed that a positive response to stimulation thresholds below 10 mA was highly suggestive of pedicle breach; but, a positive response for stimulation thresholds above 10 mA was frequently false positive [18]. Besides that, a negative response only suggests that the screw is not in proximity or abutting the nerve [10]. However, it may have violated the pedicle cortex, other than the medial wall, without troubling the nerve [19]. This was not confirmed with a routine CT evaluation. Even though such screws are not secure, they can still be considered safe, as they may not endanger neural integrity. These undetected breaches may compromise the stability of the construct and should be kept in mind [19]. However, it should be noted that, not all undetected breaches can cause symptoms. Based on our study, the symptom provoking pedicle breaches were those that breached the medial cortex, which was confirmed during the reoperation. In all such cases the screw was found to be abutting the nerve root which required repositioning. Other undetected breaches could have been present but never showed up symptomatically.

The one false negative response we encountered was perplexing as intraoperative EMG was negative but the patient had developed symptoms of neurological compromise which was later confirmed with CT, to have resulted from a malpositioned screw that had breached the medial cortex which required repositioning. This could have been avoided if intraoperative direct palpation of the medial cortex was routinely performed; however, we reserved it for those showing a positive response to t-EMG. Besides that, intraoperative perception that a screw had breached the medial cortex is not feasible always, especially if a thin layer of bone is still present over the screw [20]. Furthermore, in long constructs, we did not routinely perform a decompression favouring direct palpation of the medial cortex of the proximal most pedicle. If t-EMG had shown a positive response for such a proximal screw, redirecting the screw was first attempted. Subsequently, a laminotomy was performed to facilitate direct palpation of the medial cortex. By this way, we made sure that reoperation to reposition a malaligned screw is avoided; however, the need to attempt direct palpation or intra-operative repositioning completely depends on the t-EMG response.

Our results of sensitivity and specificity of t-EMG to detect potential pedicle breaches, is only with relation to symptomatic pedicle breaches where patients present with neurological deficits, and does not account for undetected pedicle breaches. Unlike other studies, our results showed high sensitivity for t-EMG monitoring (93.33%). This is because we had only one false negative response among 1711 screws and the false negativity of t-EMG was confirmed only with post-operative clinical examination. Various studies reported high specificity but not high sensitivity percentage; but unlike our study, they used post-operative CT to evaluate the status of the pedicles [10, 17, 21]. Hence, our analysis may be influenced by the uncertainty of true negatives due to lack of routine postoperative CT evaluation, which could have recognised more pedicle breaches, but was kept optional as the patients remained asymptomatic. Also, we only provided descriptive statistics for an analysis of stimulation threshold vs. sensitivity and specificity. However, this aspect was previously discussed in few studies [10, 22].

Considering all factors, depending solely on EMG monitoring may not be appropriate but when combined with other modalities, surgeons can benefit the most [7, 19]. Both palpatory and radiographic assessment needs to be reaffirmed by t-EMG monitoring. Furthermore, our most important finding was a significant decrease in reoperation rates with use of EMG monitoring [23]. This inference should be considered to outweigh all other uncertainties related to EMG monitoring.

Our analysis may be subject to secular influences regarding certain factors due to the retrospective nature of this study. Regarding selection of samples, we only included patients with pedicle screws among L1-S1 segments, excluding dysmorphic pedicles. However, it should be understood that, not all pedicles are anatomically similar and there can be variants or anomalies [24, 25]. The underlying pathology for which the surgery was done may have affected the pedicle anatomy. The pedicles of patients in one group may be more prone for a breach when compared to the other group. This may have influenced our analysis of reoperation rates. The overall number of screws and the number of screws per patient in Group 1 was significantly higher than that of Group 2. Besides that, our decision to use intraoperative EMG was purely based on availability. These factors may have contributed for a selection bias and could have influenced our results.

## Conclusion

We studied the efficacy of intraoperative EMG monitoring to detect potential pedicle breach during lumbar pedicle screw placement. Our results suggest that, t-EMG can be considered highly sensitive and specific for identifying potential pedicle breach by a malpositioned screw that can cause neurologic compromise; but, undetected breaches may still exist. However, t-EMG monitoring in combination with palpatory and radiographic assessment will certainly aid safe and secure pedicle screw placement. It can also efficiently reduce reoperation rates due to neurologic compromise provoked by a malpositioned screw.

## Abbreviations

CT: Computed tomography
EMG: Electromyogram
SD: Standard deviation
t-EMG: Triggered electromyogram

## References

[1] Lee CH, Hyun SJ, Kim YJ, Kim KJ, Jahng TA, Kim HJ (2014). Accuracy of free hand pedicle screw installation in the thoracic and lumbar spine by a young surgeon: an analysis of the first consecutive 306 screws using computed tomography. Asian Spine J.

[2] Oh CH, Yoon SH, Kim YJ, Hyun D, Park HC (2013). Technical report of free hand pedicle screw placement using the entry points with junction of proximal edge of transverse process and lamina in lumbar spine: analysis of 2601 consecutive screws. Kor J Spine.

[3] Seo HY, Yim JH, Heo JP, Patil AS, Na SM, Kim SK (2013). Accuracy and safety of free-hand pedicle screw fixation in age less than 10 years. Indian J Orthop.

[4] Agarwal A, Chauhan V, Singh D, Shailendra R, Maheshwari R, Juyal A (2016). A comparative study of pedicle screw fixation in dorsolumbar spine by freehand versus image-assisted technique: a cadaveric study. Indian J Orthop.

[5] Parker SL, McGirt MJ, Farber SH, Amin AG, Rick AM, Suk I (2011). Accuracy of free-hand pedicle screws in the thoracic and lumbar spine: analysis of 6816 consecutive screws. Neurosurgery.

[6] Mattei TA, Meneses MS, Milano JB, Ramina R (2009). Free-hand" technique for thoracolumbar pedicle screw instrumentation: critical appraisal of current "state-of-art. Neurol India.

[7] Puvanesarajah V, Liauw JA, Lo SF, Lina IA, Witham TF (2014). Techniques and accuracy of thoracolumbar pedicle screw placement. World J Orthop.

[8] Isley MR, Zhang XF, Balzer JR, Leppanen RE (2012). Current trends in pedicle screw stimulation techniques: lumbosacral, thoracic, and cervical levels. Neurodiagn J.

[9] Alemo S, Sayadipour A. Role of intraoperative neurophysiologic monitoring in lumbosacral spine fusion and instrumentation: a retrospective study. World Neurosurg. 2010;73(1):72–6; discussion e7. 10.1016/j.surneu.2009.04.024 20452872

[10] Parker SL, Amin AG, Farber SH, McGirt MJ, Sciubba DM, Wolinsky JP (2011). Ability of electromyographic monitoring to determine the presence of malpositioned pedicle screws in the lumbosacral spine: analysis of 2450 consecutively placed screws. J Neurosurg Spine.

[11] Flynn JM, Sakai DS (2013). Improving safety in spinal deformity surgery: advances in navigation and neurologic monitoring. Eur Spine J.

[12] Wood MJ, McMillen J. The surgical learning curve and accuracy of minimally invasive lumbar pedicle screw placement using CT based computer-assisted navigation plus continuous electromyography monitoring - a retrospective review of 627 screws in 150 patients. Int J Spine Surg. 2014;8 10.14444/1027 PMC4325487 25694919

[13] Joglekar SB, Mehbod AA (2012). Surgeon's view of pedicle screw implantation for the monitoring neurophysiologist. J Clin Neurophysiol.

[14] Pajewski TN, Arlet V, Phillips LH (2007). Current approach on spinal cord monitoring: the point of view of the neurologist, the anesthesiologist and the spine surgeon. Eur Spine J.

[15] Stecker MM (2012). A review of intraoperative monitoring for spinal surgery. Surg Neurol Int.

[16] Bose B, Wierzbowski LR, Sestokas AK (2002). Neurophysiologic monitoring of spinal nerve root function during instrumented posterior lumbar spine surgery. Spine (Phila Pa 1976).

[17] Raynor BL, Lenke LG, Bridwell KH, Taylor BA, Padberg AM (2007). Correlation between low triggered electromyographic thresholds and lumbar pedicle screw malposition: analysis of 4857 screws. Spine (Phila Pa 1976).

[18] Kulik G, Pralong E, McManus J, Debatisse D, Schizas C (2013). A CT-based study investigating the relationship between pedicle screw placement and stimulation threshold of compound muscle action potentials measured by intraoperative neurophysiological monitoring. Eur Spine J.

[19] Oner A, Ely CG, Hermsmeyer JT, Norvell DC (2012). Effectiveness of EMG use in pedicle screw placement for thoracic spinal deformities. Evid Based Spine Care J.

[20] Min WK, Lee HJ, Jeong WJ, Oh CW, Bae JS, Cho HS (2011). Reliability of triggered EMG for prediction of safety during pedicle screw placement in adolescent idiopathic scoliosis surgery. Asian Spine J.

[21] Woods M, Birkholz D, MacBarb R, Capobianco R, Woods A (2014). Utility of Intraoperative Neuromonitoring during minimally invasive fusion of the sacroiliac joint. Adv Orthop.

[22] Sharan A, Groff MW, Dailey AT, Ghogawala Z, Resnick DK, Watters WC (2014). Guideline update for the performance of fusion procedures for degenerative disease of the lumbar spine. Part 15: electrophysiological monitoring and lumbar fusion. J Neurosurg Spine.

[23] Tsai TT, Lee SH, Niu CC, Lai PL, Chen LH, Chen WJ (2016). Unplanned revision spinal surgery within a week: a retrospective analysis of surgical causes. BMC Musculoskelet Disord.

[24] Patel NP, Kumar R, Kinkhabwala M, Wengrover SI (1988). Lumbar vertebral pedicles: radiologic anatomy and pathology. Crit Rev Diagn Imaging.

[25] Amonoo-Kuofi HS (1995). Age-related variations in the horizontal and vertical diameters of the pedicles of the lumbar spine. J Anat.

